# Recent increase in *Candida auris* frequency in the SENTRY surveillance program: antifungal activity and genotypic characterization

**DOI:** 10.1128/aac.00570-24

**Published:** 2024-09-12

**Authors:** Mariana Castanheira, Lalitagauri M. Deshpande, Paul R. Rhomberg, Cecilia G. Carvalhaes

**Affiliations:** 1 Element Iowa City (JMI Laboratories), North Liberty, Iowa, USA; University of Iowa, Iowa City, Iowa, USA

**Keywords:** *Candida auris*, international clades, antifungal susceptibility

## Abstract

We observed an increase in the frequency of *Candida auris* among invasive candidiasis isolates in the 2022 SENTRY Antifungal Surveillance Program compared to prior years: ≤0.1% before 2018, 0.4%–0.6% from 2018 to 2021, and 1.6% in 2022. *C. auris* isolates were collected in seven countries, but 28 (35.9%) isolates were recovered in the USA (five states; more common in New York, Texas, and New Jersey) and 26 (33.3%) in Panama. Greece and Turkey had 12 and 9 isolates, respectively. Overall, 82.1% of the isolates were resistant to fluconazole; 17.9% were resistant to amphotericin B; and 1.3% were resistant to caspofungin, anidulafungin, or micafungin (Centers for Disease Control and Prevention tentative resistance breakpoints). Rezafungin inhibited 96.2% of the isolates (Clinical and Laboratory Standards Institute susceptibility breakpoint). Pandrug resistance was not observed, but 17.9% of the isolates were resistant to fluconazole and amphotericin B. South Asian (Clade I) isolates were most common (*n* = 40, 51.3%); of these, 97.5% were resistant to fluconazole and 30.0% were resistant to amphotericin B. Thirty (38.5%) isolates belonged to the South American region (Clade IV), and 56.7% of those were resistant to fluconazole and 6.7% to amphotericin B. Seven isolates belonged to the South African Clade III and one to East Asian Clade II. Erg11 (Y132F, K143R, and F126L) and MRR1 (N647T) alterations were detected. One isolate that was resistant to all echinocandins carried an FKS R1354G alteration. Two isolates displayed elevated rezafungin minimum inhibitory concentration (MIC) values but low MIC values against other echinocandins and no FKS alterations. As *C. auris* is spreading globally, monitoring this species is prudent.

## INTRODUCTION

*Candida auris*, an emerging fungal pathogen that can cause invasive infections, has become a significant global concern in recent years due to its dissemination potential, unique epidemiology, and resistance to multiple antifungal agents (1, 2). Originally described in 2009 from an external ear canal discharge culture of a patient in Japan (3), *C. auris* may have been causing human infections for decades. According to a retrospective study from South Korea, the earliest strain of *C. auris* was recovered from a patient with candidemia in 1996 (4). However, this organism was likely underreported. Most commercial identification systems not only were unable to provide accurate identification of *C. auris* isolates until recently but also commonly misidentified it as other yeast species such as *Candida haemulonii*, *Candida duobushaemulonii*, *Candida famata*, *Candida sake*, *Rhodotorula glutinis*, and *Saccharomyces cerevisiae* (5, 6).

*C. auris* emerged near simultaneously around the world and has rapidly spread across healthcare settings in all continents except Antarctica, posing a serious threat to patient safety and public health (7, 8). This global emergence has been attributed to the appearance of at least four geographically restricted clades, with clonal transmission identified both within and across healthcare facilities (2, 9). The clades were named according to the geographic origin of the isolates: South Asian (Clade I), Eastern Asian (Clade II), South African (Clade III), and South American (Clade IV) (2). A fifth clade has been recently described in Iran (10). These clades are genetically distinct, but within each clade, a few nucleotide differences have been observed among isolates.

Similar to other *Candida* species, *C. auris* is often associated with bloodstream infections, invasive candidiasis, and other severe infections in vulnerable patient populations, including immunocompromised patients, patients receiving immunosuppressive treatments, or patients with underlying health conditions. However, unlike other species of *Candida*, *C. auris* colonizes the skin and nares and has the ability to persist on environmental surfaces, leading to outbreaks that are challenging to control (11). Although candidemia is the most commonly observed invasive infection caused by *C. auris*, with in-hospital mortality rates reported on the order of 30%–60%, clinical *C. auris* isolates have been recovered from a variety of specimen types, including non-invasive sites such as airways, urinary and gastrointestinal tracts, wounds, and mucocutaneous swabs (9). In addition to its unique epidemiology, *C. auris* is notorious for its resistance to commonly used antifungal agents, including azoles, echinocandins, and polyenes, which are the mainstay of treatment for invasive *Candida* infections.

We observed a dramatic increase in *C. auris* isolates in the SENTRY Antifungal Surveillance Program in 2022 rising from <0.1% prior to 2018 to 1.6% of identified *Candida* spp. in 2022. In this study, we report the prevalence, susceptibility profiles, and genetic characteristics of the *C. auris* isolates submitted during this global surveillance from 2013 to 2022. We also report the activity of the recently approved long-acting echinocandin, rezafungin, and other antifungal agents when tested against these isolates.

## MATERIALS AND METHODS

### *C. auris* isolates

A total of 78 *C*. *auris* isolates were identified among 18,471 consecutive, non-duplicated invasive *Candida* clinical isolates submitted to the SENTRY Antifungal Surveillance Program from 1 January 2013 to 31 December 2022. Overall, isolates were collected in 148 hospitals located in 44 countries from bloodstream (53 isolates), skin/soft tissue (11 isolates), urinary tract (8 isolates) infections, pneumonia in hospitalized patients (4 isolates), and other or non-specified sites (2 isolates). Organism identification was confirmed by matrix-assisted laser desorption ionization-time of flight mass spectrometry using the research use only library or molecular methods as previously described (12, 13). Fifteen of the 78 *C*. *auris* isolates were previously published by Pfaller et al. (14).

### Antifungal susceptibility testing

All isolates were tested by the broth microdilution method as described by Clinical and Laboratory Standards Institute (CLSI) M27 and M2744S (15, 16). The Centers for Disease Control and Prevention (CDC) tentative resistant breakpoints for fluconazole (≥32 mg/L), amphotericin B (≥2 mg/L), anidulafungin (≥4 mg/L), caspofungin (≥2 mg/L), and micafungin (≥4 mg/L) were applied (https://www.cdc.gov/candida-auris/hcp/laboratories/antifungal-susceptibility-testing.html?CDC_AAref_Val=https://www.cdc.gov/fungal/candida-auris/c-auris-antifungal.html). Additionally, the rezafungin-susceptible (≤0.5 mg/L) provisional breakpoints published by CLSI were applied (15). These breakpoints have been confirmed by the CLSI Subcommittee of Antifungal Susceptibility Testing on 20 January 2024 and published by Locke et al. (17).

Quality control was performed by concomitantly testing *Candida parapsilosis* ATCC 22019 and *Candida krusei* ATCC 6258 according to M27 and M27M44S guidelines (15, 16). Acceptable minimum inhibitory concentration (MIC) ranges for the QC strains were published by CLSI in the M27M44S document (15).

### Whole-genome sequencing and analysis

All *C. auris* isolates were submitted to whole-genome sequencing. Total genomic DNA was used as input material for library construction prepared using the Nextera XT or Illumina DNA Prep library construction protocol and index kit (Illumina, San Diego, CA, USA) following the instructions of the manufacturer. Sequencing was performed on a MiSeq or NextSeq 1000 Sequencer (Illumina). Reads were error corrected using BayesHammer, and each sample was assembled using a reference-guided assembly in DNASTAR SeqMan NGen v.14.0 (Madison, WI, USA). Single nucleotide polymorphism-based phylogenetic analysis was performed (https://doi.org/10.1038/nmeth.4285) by comparing the study isolates to the international clades described by Lockhart et al. (2): isolates B8441 South Asian Clade I, B11220 East Asia Clade II, B11221 South Africa Clade III, B11243 South America Clade IV, and the Clade V isolate recently described (10).

## RESULTS

**Fig 1:**
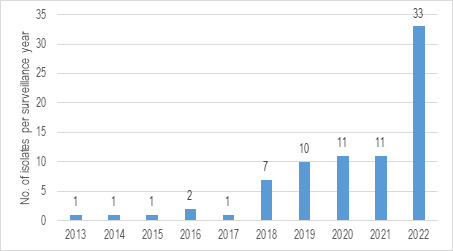
Increase of *C. auris* isolates between 2013 and 2022. *X*-axis: number of isolates per surveillance year. *Y*-axis: study year.

The overall prevalence of *C. auris* during the study period was 0.4% (78 of 18,471); however, the occurrence of these isolates increased after 2017 (Fig. 1). Before 2018, only 1 or 2 isolates were recovered during each surveillance year; these numbers increased to 7 in 2018 and then to 10 or 11 isolates yearly from 2019 to 2021. A more recent increase in the number of *C. auris* isolates was noted in 2022, when 33 *C*. *auris* isolates were submitted to the SENTRY Antifungal Surveillance Program. The percentage changes observed were from ≤0.1% of *Candida* isolates before 2018 to 0.4%–0.6% during the 2018–2021 period and then up to 1.6% (33 of 2,015 total *Candida* isolates) in 2022.

*C. auris* isolates were submitted by hospitals located in seven countries; however, 28 and 26 of the 78 isolates were collected in the USA and Panama, respectively. In the USA, *C. auris* has been observed since 2013, with a recent increase in 2022, whereas this species appeared in Panama in 2018 with similar numbers in subsequent years. Within the USA, *C. auris* isolates were more common in hospitals located in New York (13 of 28 isolates, two hospitals), Texas (8 of 28 isolates), and New Jersey (5 of 28 isolates); one isolate each also came from Indiana and Kentucky. Greece and Turkey also had elevated *C. auris* numbers compared to other surveillance regions, with 12 isolates collected in Greece since 2020 and 9 isolates from Turkey in 2022. In 2022, one isolate per country was detected in Colombia, Israel, and Japan.

Applying the resistance-only tentative breakpoints from the CDC, 82.1% of the isolates were resistant to fluconazole; 17.9% of the isolates were resistant to amphotericin B; and 1.3% were resistant to caspofungin, anidulafungin, or micafungin (Table 1). Rezafungin inhibited 96.2% of the *C. auris* isolates using the CLSI susceptible provisional breakpoint (15). Fourteen (17.9%) isolates were resistant to both fluconazole and amphotericin B. Three fluconazole-resistant *C. auris* isolates were not susceptible to rezafungin, although two of these isolates, both collected from Texas, USA, in 2022, were not resistant to other echinocandins. None of the isolates collected were resistant to all three antifungal classes.

**TABLE 1:** Antifungal susceptibility profiles of *C. auris* isolates when tested by the CLSI reference broth dilution method^*b*^

Antifungal agent	No. of isolates (cumulative %) at MIC (mg/L)															MIC_50_ (mg/L)	MIC_90_ (mg/L)	% S^*a*^	% R^*a*^
	0.015	0.03	0.06	0.12	0.25	0.5	1	2	4	8	16	32	64	128	>				
Rezafungin		1 (1.3)	4 (6.4)	9 (17.9)	43 (73.1	18 (96.2)	2 (98.7)	0 (98.7)	0 (98.7)						1 (100.0)	0.25	0.5	96.2	
Anidulafungin			2 (2.6)	4 (7.7)	44 (64.1)	23 (93.6)	4 (98.7)	0 (98.7)	1 (100)							0.25	0.5		1.3
Caspofungin	1 (1.3)	6 (9.0)	23 (38.5)	38 (87.2)	8 (97.4)	1 (98.7)	0 (98.7)	0 (98.7)	0 (98.7)						1 (100.0)	0.12	0.25		1.3
Micafungin			6 (7.7)	37 (55.1)	33 (97.4)	1 (98.7)	0 (98.7)	0 (98.7)	1 (100.0)							0.12	0.25		1.3
Fluconazole								2 (2.6)	7 (11.5)	3 (15.4)	2 (17.9)	6 (25.6)	20 (51.3)	10 (64.1)	28 (100.0)	64	>128		82.1
Amphotericin B						9 (11.5)	55 (82.1)	13 (98.7)	1 (100.0)							1	2		17.9

^*a*^ R, resistant; S, susceptible.

^*b*^ Tentative resistance-only breakpoints for anidulafungin, caspofungin, micafungin, fluconazole, and amphotericin B are published at https://www.cdc.gov/candida-auris/hcp/laboratories/antifungal-susceptibility-testing.html?CDC_AAref_Val=https://www.cdc.gov/fungal/candida-auris/c-auris-antifungal.html. Rezafungin susceptible-only breakpoints are published in the CLSI M27M44S document.

A total of 40 isolates belonged to the South Asian Clade I, including all isolates from Greece (12 isolates), all isolates from Turkey (9 isolates), and 19 isolates from the USA. Of the Clade I USA isolates, 5 isolates were from New Jersey; 13 were from New York; and 1 was from Texas (Table 2). Isolates belonging to Clade I were proportionally more resistant to fluconazole (97.5% resistant; only one isolate was susceptible) when compared to other clades (Table 3); 30.0% of Clade I isolates were resistant to amphotericin B. All Clade I isolates were susceptible to rezafungin and were not resistant to the other echinocandins tested (Table 3). Fluconazole-resistant Clade I isolates (39 of 40 isolates) had amino acid alterations previously recognized to confer resistance to this azole: Y132F (20 isolates), K143R (18 isolates), and Y132F plus K143R (1 isolate). Notably, all nine fluconazole-resistant *C. auris* isolates from Turkey carried the Erg11 alteration Y132F, while all 18 fluconazole-resistant isolates from the USA had the amino acid change K143R. Alterations in CDR1 were also noted, including a change of V704L in isolates from the USA and E709D in isolates from Greece and Turkey. The only fluconazole-susceptible Clade I isolate was collected in Texas, USA.

**TABLE 2:** Genetic analysis of *C. auris* isolates

Clade	No. of isolates	State/country	Resistance/not susceptible^*a*^	Amino acid alterations^*b*^						
				ERG3	ERG11	UPC2	CDR1	FKS1	MRR1	TACB1
Clade I: South Asia (*n* = 40)	5	Greece	FLU	Wild type	Y132F	Wild type	E709D	Wild type	Wild type	Wild type
	2	Greece	FLU	Wild type	Y132F	Wild type	E709D	Wild type	Wild type	P862R
	1	Greece	FLU	Wild type	Y132F, K143R	Wild type	E709D	Wild type	Wild type	Wild type
	2	Greece	FLU	Wild type	Y132F, K177R	Wild type	E709D	Wild type	Wildt ype	Wild type
	2	Greece	FLU, AMB	Wild type	Y132F	Wild type	E709D	Wild type	Wild type	Wild type
	4	New Jersey, USA	FLU, AMB	Wild type	K143R	Wild type	V704L	Wild type	Wild type	Wild type
	1	New Jersey, USA	FLU	Wild type	K143R	Wild type	V704L	Wild type	Wild type	Wild type
	7	New York, USA	FLU	Wild type	K143R	Wild type	V704L	Wild type	Wild type	Wild type
	6	New York, USA	FLU, AMB	Wild type	K143R	Wild type	V704L	Wild type	Wild type	Wild type
	1	Texas, USA		Wild type	Wild type	Wild type	Wild type	Wild type	Wild type	Wild type
	8	Turkey	FLU	Wild type	Y132F	Wild type	E709D	Wild type	Wild type	Wild type
	1	Turkey	FLU	Wild type	Y132F	Wild type	E709D	Wild type	Wild type	A15T, S195C
Clade II: East Asia (*n* = 1)	1	Japan	FLU	Wild type	Wild type	Wild type	E1414G	L1572I	Frameshift	D867Y
Clade III: South Africa	1	Israel	FLU	Wild type	V125A, F126L	Wild ype	Wild type	Wild type	N647T	T346I, F580L, Y647C, frameshift at 776
(*n* = 7)	1	Texas, USA	FLU	Wild type	V125A, F126L	Wild type	Wild type	Wild type	N647T	I268V, C334F, L335S, T346I, F580L, Y647C, frameshift at 776
	1	Texas, USA	FLU	Wild type	V125A, F126L	Wild type	Wild type	Wild type	N647T	T346I, F580L, Y647C, frameshift at 776
	1	Texas, USA	FLU	Wild type	V125A, F126L	Wild type	Wild type	Wild type	N647T	T346I, F580L, Y647C, frameshift at 776
	1	Texas, USA	FLU	Wild type	V125A, F126L	Wild type	Wild type	Wild type	N647T	F580L, Y647C
	1	Texas, USA	FLU, REZ	Wild type	V125A, F126L	Wild type	Wild type	Wild type	N647T	C334F, L335S, T346I, F580L, Y647C, frameshift at 776
	1	Texas, USA	FLU, REZ	Wild type	V125A, F126L	Wild type	Wild type	Wild type	N647T	T346I, Y647C, frameshift at 776
Clade IV: South America	1	Colombia	FLU	S58T	K177R, N335S, E343D, Y501H	Wild type	H771R	Wild type	Wild type	A651T
(*n* = 30)	6	Panama		S58T	K177R, N335S, E343D	A486T	Wild type	Wild type	Wild type	Wild type
	1	Panama		S58T	K177R, N335S, E343D	A486T	Wild type	Wild type	Wild type	Frameshift at 335
	6	Panama	FLU	S58T	K143R, K177R, N335S, E343D	A486T	Wild type	Wild type	Wild type	Wild type
	1	Panama	FLU	S58T	K177R, N335S, E343D	A486T	Wild type	Wild type	Wild type	L335S
	3	Panama	FLU	S58T	K177R, N335S, E343D	Wild type	H771R	Wild type	Wild type	Wild type
	1	Panama	FLU	S58T	K177R, N335S, E343D	Wild type	Wild type	Wild type	Wild type	R614K
	5	Panama		S58T	K177R, N335S, E343D	Wild type	Wild type	Wild type	Wild type	Wild type
	1	Panama	FLU	S58T	Y132F, K177R, N335S, E343D	Wild type	Wild type	Wild type	Wild type	Wild type
	1	Panama	FLU, AMB	S58T	K177R, N335S, E343D	Wild type	Wild type	Wild type	Wild type	R614K
	1	Panama	FLU, ECH	S58T	K143R, K177R, N335S, E343D	A486T	Wild type	R1354G	Wild type	Wild type
	1	Kentucky, USA	FLU, AMB	S58T	K177R, N335S, E343D	Wild type	H771R, G995S	Wild type	Wild type	Wild type
	1	Indiana, USA		S58T	K177R, N335S, E343D	Wild type	H771R, G995S	Wild type	Wild type	P862S
	1	Texas, USA	FLU	S58T	K177R, N335S, E343D	Wild type	H771R	Wild type	Wild type	L763F

^*a*^ AMB, amphotericin B; ECH, echinocandin; FLU, fluconazole; REZ, rezafungin.

^*b*^ Alterations previously described to cause resistance are underlined.

**TABLE 3:** Antifungal susceptibility profiles of *C. auris* isolates stratified by clade when tested by the CLSI reference broth dilution method

Antifungal agent	Clade I: South Asia (40 isolates)^*a*^^,*b*^					Clade IV: South America (30 isolates)^*a*^^,*b*^					Clade III: South Africa (7 isolates)^*a*^^,*b*,*c*^				Clade II: East asia (1 isolate)^*a*^^,*b*,*c*^	
	MIC_50_	MIC_90_	Range	% S	% R	MIC_50_	MIC_90_	Range	% S	% R	MIC_50_	Range	% S	% R	MIC value	Interpretation
Rezafungin	0.25	0.5	0.06 to 0.5	100.0		0.25	0.5	0.03 to >4.0	96.7		0.5	0.12 to 1.0	71.4		0.008	Susceptible
Anidulafungin	0.25	0.5	0.06 to 1.0		0.0	0.25	0.5	0.06 to 4.0		3.3	0.5	0.25 to 1.0		0.0	0.25	Not resistant
Caspofungin	0.12	0.25	0.03 to 0.5		0.0	0.06	0.12	0.015 to >4.0		3.3	0.06	0.06 to 0.12		0.0	0.03	Not resistant
Micafungin	0.12	0.25	0.06 to 0.5		0.0	0.12	0.25	0.06 to 4.0		3.3	0.12	0.12 to 0.25		0.0	0.06	Not resistant
Fluconazole	128	>128	4 to >128		97.5	64	128	2 to >128		56.7	>128	128 to >128		100.0	64	Resistant
Amphotericin B	1	2	1 to 2		30.0	1	1	0.5 to 4.0		6.7	0.5	0.5 to 1.0		0.0	1	Not resistant

^*a*^ MIC_50_, MIC_90_, range, and MIC value are in milligram per liter.

^*b*^ Tentative resistant-only breakpoints for anidulafungin, caspofungin, micafungin, fluconazole, and amphotericin B are published at https://www.cdc.gov/candida-auris/hcp/laboratories/antifungal-susceptibility-testing.html?CDC_AAref_Val=https://www.cdc.gov/fungal/candida-auris/c-auris-antifungal.html. Rezafungin susceptible-only breakpoints are published in the CLSI M27M44S document.

^*c*^ MIC_90_ values are not available for <10 isolates.

Genetic analysis demonstrated that 30 isolates belonged to the South American Clade IV. The isolates belonging to Clade IV were the 26 isolates from Panama, 3 isolates from the USA (Indiana, Kentucky, and Texas), and 1 isolate from Colombia. Fluconazole resistance among isolates belonging to Clade IV was 56.7%, and resistance to amphotericin B was 6.7%. Among the 17 fluconazole-resistant isolates belonging to Clade IV, 8 harbored the alteration K143R in Erg11 and 1 carried Y132F. One isolate from Colombia carried the Erg11 alteration Y501H that was suggested to contribute to azole resistance in association with other resistance mechanisms (18). Alterations in Erg3 or CDR1 were not consistently detected, differentiating fluconazole-resistant isolates from susceptible isolates; however, all isolates belonging to Clade IV displayed a polymorphism in Erg3 (S58T). Two Clade IV isolates were resistant to amphotericin B and one was resistant/non-susceptible to all echinocandins (MIC values ≥4 mg/L for anidulafungin, caspofungin, micafungin, or rezafungin). The latter isolate harbored an FKS1 amino acid substitution in position R1354G.

Six of the seven South African Clade III isolates were from Texas in the USA. The remaining isolate was from Israel. All Clade III isolates were resistant to fluconazole and harbored the Erg11 alteration F126L known to confer resistance to this agent; Erg11 V125A alterations were also observed in all Clade III isolates. Additionally, these isolates carried the MRR1 amino acid alteration N647T that has been demonstrated to increase fluconazole MIC values (19). Interestingly, two isolates displayed rezafungin MIC values of 1 mg/L (non-susceptible) but no FKS mutations. These isolates were not resistant to other echinocandins, but the anidulafungin MIC values (0.5 and 1.0 mg/L) were above the modal MIC for that agent for the isolates tested in this study (0.25 mg/L).

One isolate from Japan belonged to the East Asian Clade II and displayed fluconazole resistance without mutations in Erg11 or Erg3. This isolate carried an alteration in CDR1 (E1414G) and a two-nucleotide frameshift in F667 in MRR1. Additionally, this isolate harbored an amino acid substitution in FKS1 (L1572I) despite its low MIC values to the echinocandins (Table 2).

Additional analysis of CDR2, MDR1, and TAC1A demonstrated polymorphisms specific to each clade. Clade-specific alterations in TAC1B were also noted. However, certain isolates harbored alterations that were not observed in all isolates belonging to the same clade or that were noted in susceptible and resistant isolates (Table 2). The changes noted were not ones recently described to confer azole resistance (20–22).

## DISCUSSION

Since its emergence, *C. auris* has been proven to be a challenge due to its ability to disseminate, survival in the environment, and intrinsic resistance to multiple classes of antifungal agents (23, 24). Furthermore, this organism has been recognized as a priority fungal pathogen by the World Health Organization and as an urgent threat to human health by the CDC (25, 26). We observed an increase in the number of *C. auris* submitted to the SENTRY Antifungal Surveillance Program after 2018–2021 from 7–11 isolates/year to 33 isolates in 2022. Moreover, the number of countries that submitted *C. auris* isolates in our surveillance program went from three or fewer until 2021 to seven counties contributing isolates in 2022. In a surveillance of *C. auris* infections and carriage in European countries during 2020 and 2021, Kohlenberg et al. reported that bloodstream infections caused by this organism were initially reported in 2016 with a slight increase in 2017, followed by a decline in 2018 and 2019 (27). However, the authors also reported an increase of these occurrences in 2020 and 2021. These numbers were particularly driven by a large number of isolates from Spain, where *C. auris* is considered endemic, but also in Italy and Greece. Additionally, the detection of *C. auris* infections or carriage rose from 1 to 3 European countries until 2017, from 5 to 9 countries from 2018 to 2020, and 13 countries in 2021 (27). In a recent survey of *C. auris* isolates from screening and clinical cases in the USA, the CDC reported both an increase in the frequency of *C. auris* isolates and in the number of states where these isolates were reported in 2020 and 2021 when compared to 2019 (28). In our surveillance, *C. auris* isolates have been reported in New York since 2013 and in New Jersey since 2015, but in the last 2 years (2021 and 2022), three additional states submitted *C. auris* isolates. Our findings, combined with observations from the literature, confirm the global expansion of *C. auris* and the need to continue to monitor and report these isolates.

In the present study, we observed elevated fluconazole resistance rates (82.1% of the isolates); in contrast, amphotericin B resistance was noted among 17.9% of the isolates, and resistance to anidulafungin, caspofungin, and micafungin was observed in only 1.3% of the isolates. Rezafungin was active against 96.2% of the isolates. In addition, <20% of the isolates were resistant to both fluconazole and amphotericin B, and none of the isolates were resistant to all three antifungal classes. In a meta-analysis of published *C. auris* susceptibility rates, Sekyere et al. reported that 44.3% of the *C. auris* isolates were resistant to fluconazole, 15.6% were resistant to amphotericin B, 12.7% were resistant to voriconazole, and 3.5% were resistant to caspofungin (24). Notably, the resistance rates to anidulafungin and micafungin in the meta-analysis were much lower (1.3%). A similar discrepancy in echinocandin resistance rates was noted in a review by Chen et al., which reported resistance rates of 12.1% for caspofungin and 0.8% and 1.1% for anidulafungin and micafungin, respectively (29). The author reports that most caspofungin-resistant isolates were from India. Issues that arise when testing caspofungin have been thoroughly discussed in the literature (30); accordingly, resistance rates to caspofungin should be evaluated with caution due to the discrepancy with other echinocandins and the lack of FKS alterations reported in isolates that are only resistant to caspofungin (31).

We observed two isolates displaying elevated rezafungin MIC values (1 mg/L) that would be categorized as non-susceptible when applying the CLSI susceptibility breakpoint for rezafungin (≤0.5 mg/L) (15). These isolates displayed MIC values that were not categorized as resistant to other echinocandins when applying the CDC tentative resistance breakpoints (≥4 mg/L for anidulafungin and micafungin and ≥2 mg/L for caspofungin). However, their anidulafungin MIC values (0.5 and 1 mg/L) were greater than the modal MIC for the isolates tested against this agent in this study (0.25 mg/L). These isolates were collected in the same hospital in Texas, USA, and did not harbor FKS mutations. Berkow and Lockhart (32) reported the activity of rezafungin against 100 *C*. *auris* isolates, including eight isolates that were resistant to one or more echinocandins. Only four of those isolates would be categorized as non-susceptible to rezafungin, and these four isolates carried an FKS1 S639P substitution. In that study, no isolates with elevated rezafungin and low MIC values for other echinocandins were observed. Further evaluation of the isolates displaying elevated rezafungin and low MIC values for other echinocandins is under way, given the uncommon nature of this phenotype.

Genetic analysis initially identified four clades of *C. auris* (2), and one new clade has been recently reported in Iran (10). Isolates within clades are genetically closely related but are highly divergent when isolates from the distinct clades are compared. The susceptibility profiles of the *C. auris* isolates belonging to the different clades vary and—despite the numerous publications on various aspects of *C. auris* biology, infections, and treatment—susceptibility surveys from isolates recovered from multiple countries and side-by-side comparisons of the susceptibility rates among the clades are scarce. In this study, we noted that most isolates collected belonged to the South Asian (40 isolates, 51.3%) or South American (30 isolates, 38.5%) clades. Isolates from the South Asian clade were highly resistant to fluconazole (97.5%), and many were resistant to amphotericin B (30%), while isolates from the South American clade displayed lower resistance rates to both agents (56.7% and 6.7%, respectively). Additionally, the seven isolates from the South African and the single isolate from the East Asian clade were resistant to fluconazole but not amphotericin B. Echinocandin resistance was only noted in the South American and South African clades, with one (3.3%) and two (28.6%) isolates from these clades, including two rezafungin-only non-susceptible isolates from the South African clade.

The mechanisms underlying *C. auris* resistance are complex and multifaceted. They involve genetic mutations, efflux pump overexpression, and changes in cell membrane composition, all of which collectively contribute to reduced susceptibility to antifungal drugs (33). In addition, different from most other *Candida* species but similar to *C. glabrata*, *C. auris* is haploid, so a single allele mutation can lead to resistance (33).

Resistance mechanisms in *C. auris* are clade specific for the most part. We observed the Erg11 alterations Y132F and/or K143R in isolates from Clades I and IV and the F126L alteration in isolates from Clade III; these alterations are known to confer fluconazole resistance and only occur in these specific Clades (2, 18). Also, the MRR1 alteration N647T that was recently described to increase fluconazole MIC values (19) was noted in all Clade III isolates, and a frameshift of two nucleotides was noted in the Clade II isolates. Two fluconazole-resistant isolates did not have Erg11 alterations or other mutation-driven azole resistance mechanisms. Overexpression of CDR1 and MDR1 has been reported to contribute to azole resistance (34, 35), but this mechanism was not evaluated in the current study and may have been responsible for the observed phenotype. Lastly, one isolate resistant to all four echinocandins tested displayed a R1354G alteration in the FKS1 hot spot (HS)2. The substitution of an arginine by a histidine (R1354H) at the same position was recently studied by Kiyohara et al. (36). The authors reported an increase in caspofungin MIC values of 4- to 16-fold when this change was introduced in an echinocandin-susceptible isolate.

### Conclusion

In this study, we reported a significant increase in the prevalence of *C. auris* isolates in a global surveillance program. The isolates evaluated were mostly resistant to fluconazole, but amphotericin B resistance was lower or similar than other reports. In these isolates, echinocandin resistance also was very low. Rezafungin displayed good activity against 96.2% of the *C. auris* isolates. Echinocandins are recommended for the treatment of *C. auris* (37) infections and the once-weekly dosing for rezafungin could be advantageous for certain patients. In an evaluation of *C. auris* isolates in the neutropenic mouse model, Lepak et al. concluded that >90% of the *C. auris* isolates would be treatable with the recommended dose of rezafungin of 400 mg administered intravenously as a loading dose followed by 200 mg once a week (38). Hager et al. also demonstrated the potent activity of rezafungin against *C. auris* isolates in a disseminated candidiasis mouse model (39). Susceptibility profiles and epidemiology of *C. auris* isolates should be monitored since outbreaks of this pathogen often occur in healthcare facilities where patients are already immunocompromised, making the consequences of resistance-driven treatment failures potentially catastrophic. These intertwined challenges necessitate a concerted effort from healthcare providers, researchers, and public health agencies to understand, control, and combat this emerging organism.
